# Academic Well-Being and Structural Characteristics of Peer Networks in School

**DOI:** 10.3390/ijerph17082848

**Published:** 2020-04-21

**Authors:** Arja Rimpelä, Jaana M. Kinnunen, Pirjo Lindfors, Victoria Eugenia Soto, Katariina Salmela-Aro, Julian Perelman, Bruno Federico, Vincent Lorant

**Affiliations:** 1 Faculty of Social Sciences, Unit of Health Sciences, Tampere University, 33014 Tampere, Finland; arja.rimpela@tuni.fi (A.R.); pirjo.lindfors@tuni.fi (P.L.); 2 PERLA—Tampere Centre for Childhood, Youth and Family Research, Tampere University, 33014 Tampere, Finland; 3 Department of Adolescent Psychiatry, Tampere University Hospital, Pitkäniemi Hospital, 33380 Nokia, Finland; 4 PROESA, Public Health Department, Universidad ICESI, Cali, Colombia; vesoto@proesa.org.co; 5 Institute of Health and Society, Université Catholique de Louvain, 1200 Brussels, Belgium; vincent.lorant@uclouvain.be; 6 Educational Sciences, University of Helsinki, 00014 Helsinki, Finland; katariina.salmela-aro@helsinki.fi; 7 Escola Nacional de Saúde Pública, Universidade Nova de Lisboa, 1600-560 Lisboa, Portugal; jperelman@ensp.unl.pt; 8 Department of Human Sciences, Society and Health, University of Cassino and Southern Lazio, 03043 Cassino, Italy; b.federico@unicas.it

**Keywords:** adolescents, peers, school burnout, schoolwork engagement, social network analysis

## Abstract

Peer networks at school and students’ position in these networks can influence their academic well-being. We study here individual students’ network position (isolation, popularity, social activity) and peer network structures at the school level (centralization, density, clustering, school connectedness) and their relations to students’ academic well-being (school burnout, SB; schoolwork engagement, SE). Classroom surveys for 14–16-year-olds (*N* = 11,015) were conducted in six European cities (SILNE survey). Students were asked to nominate up to five schoolmates with whom they preferred to do schoolwork. SB and SE correlated negatively (−0.32; *p* < 0.0001). Students had on average 3.4 incoming (popularity; range 0–5) and 3.4 outgoing (social activity; 0–5) social ties. Percentage of isolated students was 1.4. Students’ network position was associated weakly with academic well-being—popular students had less SB and higher SE, and socially active students had higher SE. School-level peer networks showed high clustering and school connectedness, but low density and low centralization. Clustering was associated with higher SB. Low centralization and high school connectedness protected from SB. Dense networks supported SE as did high average school connectedness. Correlations between these network indicators and academic well-being were, however, low. Our study showed that both students’ network position and network characteristics at the school level can influence adolescents’ academic well-being.

## 1. Introduction

The school context constitutes an important social environment where adolescents are connected to each other on a daily basis. In general, the school context has a major influence on pupils’ general subjective and academic well-being, academic achievement, mental and physical health, and even their lifestyle [1,2,3,4,5]. Orientation to peers and friendships is one of the key features of adolescence, and through these interactions adolescents absorb a wide range of attitudes, norms, and experiences and skills [6,7,8]. Peer interactions among adolescents are complex and multidimensional and can take many forms, from best friend dyads to large friendship groups [9,10,11,12,13].

Previous research has shown that peer group context has various effects on adolescents’ behaviours. Members of the peer groups resemble each other in many characteristics, such as alcohol use and smoking [14,15,16], and depressive symptoms [17]. This similarity of peers also extends to educational expectations [18], school engagement [19,20], and academic achievement [21,22].

In general, both positive and negative emotional school engagement seem to have remarkable interconnections with academic and psychological functioning [23,24,25,26]. In this paper, we focus on schoolwork engagement and school burnout, two indicators of students’ academic well-being [27,28]. Schoolwork engagement, which can be thought of as a narrower concept of engagement in the school context [24,29], includes energy at schoolwork, dedication toward schoolwork, and perceiving it as meaningful, as well as absorption in schoolwork so that time passes quickly when studying [26,30].

School burnout, on the other hand, is a concept related to school ill-being. Burnout has originally been regarded as a work-related disorder [31], and the concept has recently been found useful and transferrable to the school setting [32,33,34]. Thus far, research has identified many risk factors for school-related burnout, such as school pressure, peer groups, school disengagement, as well as maladjustment, thus showing the importance of the phenomenon [34]. School-related burnout can be defined as a combination of exhaustion at schoolwork, cynicism toward the meaning of school, and sense of inadequacy as a student [35]. It can be caused by discrepancies between student’s internal resources, school workload, and expectations of school results, which all can be considered to be critical signs of emotional disengagement [26,32]. According to the demands–resources model, which includes energetic and motivational aspects, the demands of a high level of study, such as stress, are related to school burnout, whereas study-related resources, such as self-efficacy, are related to engagement with schoolwork [35].

Academic well-being, including school burnout and schoolwork engagement, may also be considered from a relational perspective. In their longitudinal study, Kiuru et al. [32] examined the extent to which peer group similarity in school burnout was based on peer group influence and peer group selection. Their results showed that the group members were somewhat similar in terms of burnout, thus indicating group similarity in how group members think and feel about their school. Their results also revealed that the group similarity was due to peer group influence, a tendency to reinforce similar attributes among peer group members. Further, they showed that a membership in a high achieving peer group buffered against the increase in school burnout whereas low performing groups promoted an increase in school burnout. This suggests the need to consider adolescents’ social position in the peer group.

Adolescents’ peer networks are not random, neither at the level of the adolescent (hereafter, ego level) nor at the school level (hereafter, network level). At the ego level, some adolescents are more central than others, for instance they receive more friendship nominations compared to others. This unequal structural position has been shown to affect substance use [13]. It may also affect their academic well-being as it signals their social standing in the school. At the network level, the peer network may display different properties. The network may provide adolescents with more opportunities to reach out to different peers (or alters) and to share common norms. Alternatively, it may strengthen the cohesion and the trust in the network if friends’ friends are also friends. These network features have been shown to affect the prevalence of substance use in adolescents, beyond the ego level [36,37].

Thus far, little is known on how the structures of peer networks in school influence students’ academic well-being, especially at the school level. We aimed to improve this understanding by exploring the association between adolescent academic well-being and the characteristics of peer networks both at the school and individual levels. We used cooperation ties as a distinction from friendship ties, which are most often studied in adolescent social network research. Cooperation ties refer to social ties with those classmates with whom students prefer to do school assignments or from whom they prefer to ask help. We assumed that cooperation ties are more essential for academic well-being than friendship ties, which are based on emotional meanings and actions outside school. We asked two research questions:

1. Is student’s academic well-being related to his/her position in the peer network?
2. Is student’s academic well-being related to the peer network structure of his/her school?

## 2. Materials and Methods 

### 2.1. Participants and Study Procedure

We used data collected between January and November 2013 with the SILNE survey including six European countries. In each country, a city was selected on the basis of population size, income, and employment rates close to the national average. The cities of Namur (Belgium, BE), Hanover (Germany, GE), Tampere (Finland, FI), Latina (Italy, IT), Amersfoort (the Netherlands, NL), and Coimbra (Portugal, PT) were selected. These cities are described in Table A1. In each city, schools were selected from the local register of schools, from two socio-economic strata, and were invited to participate. School stratification was achieved on the basis of information specific to each country—either by the type of school (Italy, Germany, and the Netherlands), by the socio-economic ranking of the school by the educational authorities (Belgium and Portugal), or according to the area’s socio-economic status (Finland) (Table A2). The number of the participating schools was eight in Amersfoort (NL), Tampere (FI), and Latina (IT); seven in Namur (BE); six in Coimbra (PT); and 13 in Hanover (GE).

From each school, students in the two grades nearest to the age of 14−16 years were invited to participate. The number of participants was 11,015. Questionnaires were administered during regular school hours with paper-and-pencil method and were enclosed into envelopes immediately after filling them. Students were informed about the purpose of the study. Participation was voluntary. Ethical approvals were obtained in each country. The overall response rate was 79.4%. The response rate (number of participants) was in Namur (BE) 90% (2133), in Tampere (FI) 86% (1499), in Hanover (GE) 66% (1476), in Latina (IT) 77% (2085), in Amersfoort (NL) 81% (1922), and in Coimbra (PT) 79% (1900) (Table A2). Participants’ age varied from 12 to 19 years, with a mean of 15.24 years and a median of 15 years. The number of students who were 12, 13, 18, and 19 years old was only 424 (3.8%), and 17 year olds were 932 (8.5%) in number. All age groups were kept in the analyses. The number of students with missing information on age was 81. Of all participants, 51.9% were girls and 48.1% were boys. More information on SILNE survey procedure can be found elsewhere [38].

### 2.2. Instruments

#### 2.2.1. Well-being Measures

*Schoolwork engagement* was estimated with a short version of the originally nine-item-long Schoolwork Engagement Inventory (EDA) [30] capturing all the three dimensions: (1) absorption (“Time flies when I am studying”), (2) energy (“I feel strong and vigorous when I am studying”), and (3) dedication (“I am enthusiastic about my studies”). It was scored on a six-point scale (0 = “Never” – 5 = “Daily”). The Cronbach’s α reliability for the scale was 0.76, which was somewhat lower than the Cronbach’s α of the original inventory (0.94) [30] but still good. The mean of the three items was used to measure schoolwork engagement per adolescent. Only those participants who answered all three items were included in the analysis, meaning 3.6% were missing.

*School burnout* was assessed with the Short School Burnout Inventory (SSBI), which was developed on the basis of the nine-item-long School Burnout Inventory (SBI) [33]. The scale used in this survey contained three items measuring all the three dimensions: (1) sense of inadequacy at school (“I often have feelings of inadequacy in my schoolwork”), (2) cynicism toward the meaning of school (“I feel that I am losing interest in my schoolwork”), and (3) exhaustion at school (“I brood over matters related to my schoolwork a lot during my free time”) measured with a six-point scale (1 = “Completely disagree” – 6 = “Completely agree”). Cronbach’s α reliability for the three-item scale was 0.379, and the reliability between item 3 and the first two was especially low and, for one country, even negative (−0.013). However, the Cronbach’s α reliability for the items 1 and 2 was 0.62, which is acceptable although much lower than in the original inventory (0.88) [33], and thus they were used in this study to measure school burnout. Mean of the scores of these two items was used to measure school burnout per adolescent. Only those participants who answered both two items were included in the analysis, meaning 3.2% were missing.

These schoolwork engagement and school burnout measures were used the same way in one previous paper [39]. The shorter versions of the Schoolwork Engagement Inventory and School Burnout Inventory were used in the survey due to lack of space in the questionnaire.

#### 2.2.2. Network Measures

The social network survey used a student directory to ask about the students’ social ties at school, except for Finland where name generator was used. Student directory contained the first names and family names of all students enrolled in the two grades surveyed. The names were coded and listed alphabetically by class and grade. Participants were asked to nominate a maximum of five schoolmates with the question, “Which schoolmates do you prefer to work with or ask for advice, for example on homework or an assignment?” (later labelled “cooperation”). After that, they wrote the corresponding codes into their questionnaire. The social network survey has been described in detail elsewhere [38].

Social network measures were computed at the ego level (student level) and at school network level, where school refers to the students at the two grades included in the survey.

At the ego level, we focused on student position in the network and computed three measures to capture how important or influential a student is in the peer collaboration network [40]:

(1) *Social activity* (outgoing social ties) measure is the simplest indicator of a student connectivity with other students in their school peer network. It assigns an importance score on the basis of the number of outgoing social ties held by each student. This measure tells us how many outbound links or nominations (out of five asked in the questionnaire) each student had to other students in the network (all students who were registered in the two grades surveyed in the school).
(2) *Popularity* (incoming social ties) measure is estimated in the same way as social activity, however, we looked at the number of inbound links or nominations. This means that the number of inbound links or nominations (out of five asked in the questionnaire) each student had to other students in the network (all students who were registered in the two grades surveyed in school) was estimated.
(3) *Isolated students* is measured as the proportion (%) of students in the network (all students who were registered in the two grades surveyed in school) without reported social ties (no nominations in the questionnaire).

At the school peer network level, we focused on the structure of the school peer network composed by all students registered in the two selected grades:

(1) *Density of the school network* is a ratio of the total number of relational ties (nominations) at each school divided by the total number of potential relational ties at that school. In our case, density is the proportion of the number of student cooperation ties divided by the total possible number of cooperation ties in the student peer network, which corresponds to all possible ties that students registered in the two grades in the school can establish. It is expressed here as percentages. Zero means no ties between students and 100 means that every student has nominated all other students. A dense network at school, meaning more social ties between students, is generally associated with a higher provision of social support, a faster circulation of ideas and innovations, and a higher enforcement of norms [41].
(2) *Centralization* measure identifies the most prominent individuals in the network, which is those who are extensively involved in social relationships with other individuals in the network. In other words, it assesses the tendency of a few students in the network to have more social ties compared to others. It is expressed here as percentages. Zero means that all students have the same number of nominations and 100 means that one student is the only one to be connected to all other students. Centralization is a measure of inequality in a network and it is generally associated with better coordination and efficiency but lower satisfaction [41].
(3) *Clustering* is the average density of ties around the egos (individual students). It measures how much an ego’s friends’ friends are also the ego’s friends. It is expressed here as percentages ranging from 0 to 100. Clustering captures the network closure and the overall tendency of a network to be patterned as a small world structure—a network of several small and dense student groups connected to each other by bridges. It is computed as the average density of ties in the ego’s networks. In our study, clustering measures the proportion of one’s schoolmates (with whom one wants to do schoolwork) that are also schoolmates with each other.

*School connectedness* is a subjective measure of the bonds the students feel towards their school. It is the student’s perception of school climate, and it represents their impression of and relationship with the school, and it is highly related to the social context within which they are involved. School connectedness captures the subjective perspective about the social ties at school [42]. It is estimated with five items: (i) whether adolescents feel close to people from their school, (ii) whether they feel happy at their school, (iii) whether they feel part of their school, (iv) whether the teachers treat them fairly, and (v) whether they feel safe at school, with four options each. According to the students’ responses, a value was given per item as follows: 1 = “strongly disagree”, 2 = “disagree”, 3 = “agree”, 4 = “strongly agree”. Then, a mean value of five items was calculated per student. Next, a group mean value, ranging from 1 to 4, was estimated for each school on the basis of the answers by all participated students. This score was used in the data analysis. The scores between schools ranged from 2.42 to 3.45. 

#### 2.2.3. Controlling Variables

Socioeconomic variables were included in the analyses as controlling variables. The Family Affluence Scale (FAS) was used to measure adolescents’ socioeconomic status (SES) with four questions [43]. Additionally, age, sex, and country were used as controlling variables.

### 2.3. Data Analysis

The outcomes, school burnout, and schoolwork engagement were analysed separately. The associations with individual and school network indicators were investigated first with correlation analysis. This descriptive analysis allowed us to examine the association between the network measures and the academic well-being variables. Next, multilevel bivariate linear regressions and then multilevel multivariate linear regressions were conducted on individual and school network measures, adjusting for socioeconomic status (SES) of students, age and sex, and with school as random coefficient (nested by country). Given that the participating schools in the SILNE survey vary by SES, cultural tracks, and educational tracks (Table A1 and Table A2), we used these variables as controlling variables in order to separate their effects from individual position and school peer network, which are the variables under study. Before we performed the regression analyses, we tested the multicollinearity. Pearson’s correlation coefficients were estimated for all explanatory variables. No strong pairwise correlations were found (data not shown). We also estimated the variance inflation factor (VIF), which is a valid test to check for multicollinearity. For both models for school burnout (SB) and schoolwork engagement (SE), VIF was lower than 3. Thus, there was no multicollinearity between explanatory variables. We also provide the results from the goodness of fit test (Log-likelihood test) for the multivariate regression models.

## 3. Results

Table 1 presents the descriptive statistics and the correlations with the well-being indicators. On average, one adolescent had 3.4 incoming and 3.4 outgoing nominations. Only 1.4% had no incoming or outgoing nominations (isolated). At the school level, the networks had a low density (less than 2%) and low centralization (3.3%). The clustering coefficient was higher than 40.17%, which means that the adolescents’ networks were quite cohesive. School connectedness was quite high, with 87% of the adolescents agreeing that they felt close, happy, or part of their school, or that they felt safe or treated fairly at school. Only 13% disagreed. The average score was high—3.04 out of a maximum of 4. However, school burnout was not low, with an average score of 3.08 out of 6, whereas schoolwork engagement was quite low with 2.09/5.

The correlations of the indicators of student network position with academic well-being were of small magnitude (Table 1). Of these indicators, being isolated did not correlate with school burnout or schoolwork engagement, but popular individuals reported slightly less often school burnout and were more engaged in schoolwork (Table 1). 

The school network level indicators had only weak correlations with school burnout and schoolwork engagement (Table 1). The higher density of ties correlated positively with school burnout and negatively with schoolwork engagement. Increase in clustering of ties in the school network increased both school burnout and schoolwork engagement. School burnout was lower and schoolwork engagement higher in schools with higher school-level connectedness (Table 1.)

Table 2 and Table 3 provide the results of the multilevel linear regressions, both bivariate and multivariate models, of both academic well-being indicators on student and school network structures. 

Overall, there were few small effects of student network position on school burnout (Table 2). School burnout decreased slightly with popularity and social activity of the student in bivariate models but only with popularity in multivariate models, although with a quite small size effect (β = −0.07). In the multivariate model, school level network indicators were related to school burnout and thus higher centralization was related to a lower level of school burnout, whereas higher clustering was related to higher school burnout. School burnout strongly decreased when the school average level of connectedness increased (β = −0.59) (Table 2).

The results for schoolwork engagement are presented in Table 3. When the students’ own position showed higher popularity and higher social activity, his/her schoolwork engagement was slightly higher. At the school level, however, students’ schoolwork engagement was higher when the network density was higher. Higher school connectedness was strongly associated with higher schoolwork engagement.

## 4. Discussion

Our study is the first to investigate the relationship of cooperation social ties and academic well-being using both individual and school-level characteristics of peer networks. Our interest was whether characteristics of a student’s network position or a structure of social ties at school influence students’ academic well-being measured by context-specific well-being indicators, namely, school burnout and schoolwork engagement. The results showed that the students’ network position as well as the peer network characteristics at the school level are associated with students’ academic well-being, although the effects were small and different for the two well-being indicators. As far as the school peer network level is concerned, our study suggests that the process of school connectedness is a key attribute of the student network that is associated with academic well-being.

Thus far, most studies on adolescents’ social networks and health-related issues have analysed friendship ties, which indicate more emotional than instrumental relationships between individuals. We asked, instead, students to nominate schoolmates they preferred to do schoolwork with. These cooperation ties refer to a tendency to be a mate with those who are useful in terms of academic achievement, having a more instrumental than emotional meaning. It has been suggested that peer group processes work differently across behaviours and characteristics, for instance peer group similarity seems to be larger in visible behaviours and shared activities such as smoking, alcohol consumption, or external problem behaviours than in non-visible individual attributes such as attitudes, values, or personality [44,45,46]. Students’ school burnout and engagement in schoolwork as such are less visible to others, which may partly explain why the influence of the structures of social ties was not so high. On the other hand, measuring friendship ties may have given stronger associations with school burnout due to its more emotional nature.

The studied associations were stronger for the school peer network characteristics than for the student’s network position in both school burnout and engagement in schoolwork. This was to some extent unexpected because usually individual position in the network explains much more of the variance than school peer characteristics. Concerning school burnout, this was shown in an earlier study by Salmela-Aro et al. [47].

Of school network characteristics, school connectedness was related to both well-being indicators, and thus in schools with higher connectedness, students had less school burnout and more engagement in schoolwork. School connectedness captures the subjective perspective of social ties at school [42]. This can be interpreted as greater ease in cooperating with other students and teachers, which increases the perception of students in terms of having a positive school climate, thus leading to higher engagement in schoolwork. On the other hand, this may increase social support, which decreases the risk of school burnout [47]. Previous research has not studied school connectedness on a school peer network level and its association with context-specific school well-being indicators, but our results are in line with previous research on the association between school connectedness and various positive health and learning related outcomes at a student level [42,48,49]. 

Recent research also shows that knowledge-oriented socio-digital participation, that is, using digital tools to gain and share knowledge, may increase study engagement and thus academic well-being [50]. The other forms of socio-digital participation, social networking, and action gaming may instead decrease study engagement and increase burnout [50]. All in all, enhancing school connectedness in terms of shared values, norms, or pedagogical understanding among school teaching and other staff, and sharing these with students, may enhance students’ academic well-being.

In addition to school connectedness, increase in the network density, meaning more connections between students, supported engagement in schoolwork. Dense networks are generally associated with higher provision of social support and faster circulation of ideas and innovations [41], explaining the mechanism behind our result. On the other hand, density of the student peer networks was not related to school burnout, whereas higher clustering increased risk of school burnout and lower centralization protected against it. Clustering captures the network closure, meaning a network of several small and dense student groups connected to each other by bridges [40]. Centralization is a measure of inequality in a network when a few students have more nominations compared to others. The mechanism behind this association could not be revealed with our data, but less social support in the clustered networks and more in the less centralized ones may explain these associations. It has been shown that support from school members is negatively associated with school burnout [47].

Compared with the strength of the association between school connectedness and the well-being indicators, the associations with school network density, clustering, and centralization were weak. The difference between these measures and school connectedness is that school connectedness measures students’ perception of social relationships within the school community, whereas density, clustering, and centralization measure the actual social relationships between students. This implies that the perception of the nature of social relationships might be more important than the actual ones. In addition, the measure of school connectedness also covered relationships with teachers and was thus wider than the other measures.

Previous studies have shown the importance of peer groups in students’ school burnout and schoolwork engagement. A total of 6% of the variation in school burnout and 20% in schoolwork engagement have been shown to be due to differences between peer groups [51], meaning that students in the same group resemble each other (group homogeneity). Peer group selection and peer influence are the two mechanisms that lead to peer group similarity. In their longitudinal study, Kiuru et al. [32] found that peer influence, but not peer group selection, explained students’ burnout. Our study was cross-sectional, thus we could not study the mechanisms of peer selection or peer influence. However, we could show how a student’s position in his/her ego network was associated with well-being indicators—socially active students were more engaged in schoolwork and had a lower risk for school burnout, as was also the case for popular students. Previous studies have not concerned well-being indicators but instead have focused on other issues, for example health-compromising behaviours such as smoking, showing that popular students smoke more often [52] and that smoking increases with the popularity of smokers at school [53]. From the health point of view, opposite mechanisms of popularity operate in health-compromising behaviours and school burnout.

In our study, the correlation between school burnout and schoolwork engagement was low, which shows that they measure different dimensions of academic well-being. Tuominen-Soini et al. [28] have also presented them as two different dimensions. Thus, it was not a surprise that peer network characteristics, which protect from school burnout, were different from those that supported engagement in schoolwork. However, if we take a person-oriented approach, there is one group, namely, success-oriented students, who have high schoolwork engagement but also high school burnout as they have stronger concern of validating their competence [28].

Majority of adolescent social network research and health-related subjects have concentrated on individual (ego)-level indicators of social ties whereas less have studied network structures at the school level. In our study, we had indicators on both levels, which was innovative and showed the importance of both levels. A strength of the current study is that we analysed the network dynamics of school burnout and schoolwork engagement simultaneously. Recent research [26,28,54] has demonstrated that school burnout and schoolwork engagement are not simply the opposites of the same continuum, but distinct psychological processes contributing independently to academic and psychological outcomes—an engaged student can be burned-out at the same time. Most studies have concentrated on one behaviour or phenomenon at a time. 

Our study was conducted in six countries with diverse cultural settings, and there was large variation in the levels of school burnout and schoolwork engagement between countries. One limitation of our study was that, for school burnout, we could use only two of the three items in the Short School Burnout Inventory, and thus we cannot exclude the possibility of a compromised validity of the survey instruments. A second limitation was that our study was cross-sectional, and thus we cannot make inferences on causality.

## 5. Conclusions

Our study showed that both students’ network position and school peer network characteristics might be associated with adolescents’ academic well-being, even though the associations are small. The new finding was that not only the individual student’s network position but also the structure of the school peer network, especially school connectedness, can influence students’ academic well-being. This means that actions and interventions that promote positive school climate, good relationships between members of school community, and practices that enhance sense of belonging may contribute to students’ academic well-being.

## Figures and Tables

**Table 1:** Mean or percentage (%) and standard deviation (Std) of students’ socio-demographic and academic well-being indicators, students’ network position and school network characteristics, and their correlation with academic well-being indicators.

Covariate	Mean or %	Std	Number of Students	Pearson Correlation with	
				School Burnout	Schoolwork Engagement
Sociodemographic					
Sex, female %	51.72	49.97	10,933	0.03 **	0.12 ***
Age, years	15.24	1.05	10,934	0.01	−0.04 ***
Academic well-being					
School burnout (range 1–6)	3.08	1.24	10,659		−0.32 ***
Schoolwork engagement	2.09	1.43	10,615	−0.32 ***	
(range 0–5)					
Student network position					
Social activity, mean of	3.43	1.45	11,015	−0.01	0.06 ***
outgoing nominations					
(range 0–5)					
Popularity, mean of	3.43	2.25	11,015	−0.06 ***	0.07 ***
incoming nominations					
(range 0–5)					
Isolated students, %	1.40	11.8	10,585	0.01	0.00
School network					
characteristic					
Density, %	1.56	1.12	11,015	0.05 ***	−0.03 **
Centralization, %	3.33	0.73	11,015	−0.03 **	0.01
Clustering, %	40.17	7.14	11,015	0.08 ***	0.03 **
School connectedness	3.04	0.19	11,015	−0.06 ***	0.11 ***
(range 1–4)					

*** *p* < 0.01; ** *p* < 0.05.

**Table 2:** Standardized betas (β) and 95% CI for school burnout from the multilevel linear regression models on students’ network position and school network characteristics.

Covariate	Bivariate Models		Multivariate Model ^1^	
	β	95% CI	β	95% CI
Student network position				
Social activity	–0.02 **	–0.04, –0.00	0.01	–0.02, 0.03
Popularity	–0.07 ***	–0.09, –0.05	–0.07 ***	–0.10, –0.05
Isolated students	0.01	–0.01, 0.03	0.01	–0.01, 0.03
School network characteristic				
Density	0.02 **	0.00, 0.05	0.02	–0.01, 0.05
Centralization	–0.02	–0.04, 0.00	–0.07 ***	–0.09, −0.04
Clustering	0.03 ***	0.00, 0.06	0.03 *	–0.00, 0.06
School connectedness	–0.43 ***	–0.62, –0.24	−0.59 ***	–0.80, –0.38

^1^ Controlled for socioeconomic status (SES), age, and sex with a random coefficient at the school level. Goodness of fit test of the multivariate model: –2 log likelihood = 28,417.6. *** *p* < 0.01; ** *p* < 0.05; * *p* < 0.1.

**Table 3:** Standardized betas (β) and 95% CI for schoolwork engagement from the multilevel linear regression models on students’ network position and school network characteristics.

Covariate	Bivariate Models		Multivariate Model ^1^	
	β	95% CI	β	95% CI
Student network position				
Social activity	0.06 ***	0.04, 0.08	0.03 ***	0.01, 0.05
Popularity	0.07 ***	0.05, 0.08	0.03 ***	0.02, 0.04
Isolated students	0.00	−0.01, 0.02	0.10	−0.12, 0.31
School network characteristic				
Density	0.01	−0.01, 0.03	0.04 ***	0.02, 0.07
Centralization	–0.05 ***	−0.07, −0.03	–0.01	–0.03, 0.02
Clustering	–0.01	−0.04, 0.01	–0.02	–0.05, 0.01
School connectedness	0.84 ***	0.66, 1.02	0.85 ***	0.65, 1.04

^1^ Controlled for SES, age, and sex with a random coefficient at the school level. Goodness of fit test of the multivariate model: −2 log likelihood = 27,381.8. *** *p* < 0.01.

## Appendix A

**Table A1:** Socioeconomic characteristics of the cities surveyed in the six countries in 2013 [38].

Country	City	Description of the City	Average Income of the Country (€)	Average income of the region	Unemployment Rate in the Country	Unemployment Rate in the City (%)	Population of the City
**Belgium**	Namur	Capital of the	18,301	18,785	7.6	12.6	108,950
		province of					
		Namur and of					
		Wallonia;					
		important					
		commercial and					
		industrial					
		centre;					
		products:					
		machinery,					
		leather goods,					
		Metals, and					
		porcelain.					
**Finland**	Tampere	Third largest	25,500	25,000	9.7	12.8	215,168
		city; centre of					
		leading-edge					
		technology,					
		education,					
		research,					
		culture,					
		sports, and					
		business.					
**Germany**	Hanover	Capital of the	30,360	34,308	8.1	9.2	514,137
		federal state					
		of Lower					
		Saxony;					
		major centre					
		in northern					
		Germany;					
		hosting					
		annual					
		commercial					
		trade fairs.					
**Italy**	Latina	Capital of the	22,891	20,487	8.4	10.6	118,612
		province of					
		Latina in the					
		Lazio region;					
		pharmaceutical					
		and chemical					
		industry;					
		important					
		agricultural					
		centre.					
**The**	Amersfoort	Second largest	23,400	24,900	6.4	6.4	148,250
**Netherlands**		city of the					
		province of					
		Utrecht; one of					
		the largest					
		railway					
		junctions in					
		the country.					
**Portugal**	Coimbra	Main city of	12,408	12,348	12.7	10.0	143,396
		the Centre					
		Region; main					
		activities are in					
		the fields of					
		commerce,					
		public					
		administration,					
		education,					
		Health, and					
		social services.					

**Table A2:** Selected grades, school selection and stratification, number of schools recruited and adolescents participating, and participation rate per city in 2013 [38].

City (Country)	Selected Grades	School Selection and Stratification	Number of Schools Recruited	Number of Adolescents Participating	Participation Rate (%)
**Namur**	Last grade of lower	Based on the	7	2133	89.8
**(BE)**	secondary and	parental SES			
	first grade of upper				
	secondary education				
	(third and fourth); early				
	educational tracking				
	(at the age of 12)				
**Tampere**	Last two grades of lower	Based on the	8	1499	86.0
**(FI)**	secondary education	average income			
	(eighth and ninth); later	and proportion of			
	educational tracking	highly educated			
	(at the age of 16)	in the area			
**Hanover**	Last two grades of lower	Based on the	13	1476	66.0
**(GE)**	secondary education	average income			
	(eighth and ninth); early	in the area and			
	educational tracking	tracking/school			
	(at the age of 10)	types			
**Latina**	First two grades of upper	Based on school	8	2085	76.5
**(IT)**	secondary education	type			
	(9th and 10th); later				
	educational tracking				
	(at the age of 14)				
**Amersfoort**	Last two grades of lower	Based on the	8	1922	80.9
**(NL)**	secondary education	available school			
	(third and fourth); early	tracks in the			
	educational tracking	school			
	(at the age of 12)				
**Coimbra**	First two grades of upper	Based on the	6	1900	78.9
**(PT)**	secondary education	average income			
	(10th and 11th); later	in the area and			
	educational tracking	school type			
	(at the age of 15)				
